# Continuous glycemia monitoring in perioperative period in patients undergoing total knee or hip arthroplasty: A protocol for a prospective observational study

**DOI:** 10.1097/MD.0000000000031193

**Published:** 2022-10-21

**Authors:** Olga Adamska, Artur Mamcarz, Marcin Łapiński, Kuba Radzimowski, Piotr Stępiński, Jakub Szymczak, Maciej Świercz, Krystian Żarnovsky, Bartosz M. Maciąg, Artur Stolarczyk

**Affiliations:** a Orthopaedic and Rehabilitation Department, Medical University of Warsaw, Warsaw, Poland; b ProfTit, 3rd Clinic of Internal Medicine and Cardiology, Medical University of Warsaw, Warsaw, Poland.

**Keywords:** continuous glycemia monitoring, diabetes mellitus, diabetic complications, orthopedic surgery, orthopedics, surgery, total joint replacement

## Abstract

In every surgical subspecialty surgical site infection (SSI) or implant infections, although occur seldom, pose a threat to patients’ health. Risk factors of such states is diabetes mellitus (DM), considered one of the most widespread health-related problems of the 21st century. Orthopedists perform big joint replacements that usually concern older adults and therefore often deal with patients suffering from comorbidities. DM is frequently one of them and can furthermore often remain underdiagnosed. The other risk for complication is a rapid beginning of the rehabilitation which starts on the day following the surgery. To eliminate the debilitating impact of DM and hypoglycemia on surgical patients, we aim to investigate the relationship between the glycemia values and the postoperative outcomes in certain periods of time in patients undergoing orthopedic surgeries. Participants meeting inclusion criteria will have inserted a glycemia measuring device (Dexcom G5, Inc., San Diego, CA) in the periods of time. First time it will take place 14 days prior to the surgery and right after the surgery for the second time for the period of another 14 days. All patients will undergo standard total knee arthroplasty or total hip arthroplasty procedures. Patients will be assessed preoperatively and 14 days, 1, 3, 6, 12, and 24 months postoperatively. The assessment of the joint condition will consist of: patient-reported outcomes (The Knee injury and Osteoarthritis Outcome Score, Harris Hip Score, the Western Ontario and McMaster Universities Osteoarthritis Index [WOMAC]); assessment of potential SSI and cardiovascular complications (the Finnish Diabetes Risk Score [FINDRISC], the Systematic Coronary Risk Evaluation [SCORE]) and the clinical examination. To investigate the influence of orthopedic surgery (anesthesia) on glycemia and the significance and safety of early patients mobilization after the big joints surgeries. To investigate changes of glycemia in patients with normal glycemia metabolism, potentially protecting them from hypoglycemia during hospital stay and increasing their awareness of potential development of DM in the future. Additionally, this study will correlate perioperative glycemic levels with risk of cardiovascular events in one year follow-up, and its influence on SSI and implant complications.

## 1. Introduction

In every surgical subspecialty surgical site infection (SSI), although occur seldom, pose a serious threat to a patients’ health.^[1,2]^ Among the most known risk factors of such states are glucose metabolism disorders, which including diabetes mellitus (DM), are considered one of the most widespread health-related problems of the 21st century.^[3,4]^ DM is a civilization disease, expected to reach almost 600 million people by 2030^[5,6]^ Hyperglycemia is a potentially reducible risk factor for a variety of complications after a surgical procedure, including infections.^[7]^ Both the World Health Organization and U.S. Centers for Disease Control and Prevention recommend perioperative glucose control,^[8,9]^ but there is debate over which surgical patients should be treated and what protocol would maximize benefits and eliminate side effects. Recently, a stronger link between postoperative hyperglycemia and adverse outcomes in patients without diabetes than in patients with diabetes has become evident, suggesting that patients without diabetes may benefit especially from glucose control.^[10]^ Many patients undergoing orthopedic surgery may have unrecognized diabetes or may develop stress-related hyperglycemia in the hospital. The challenge is to minimize the effects of metabolic derangements on surgical outcomes, reduce glycemic excursions, and prevent hypoglycemia.^[11–13]^ There are no clear guidelines advocating evidence-based glucose targets in the inpatient setting and regimens. Individualized treatment should be based on the ambient level of glycemic control, outpatient treatment regimen, presence of complications, nature of the surgical procedure, and type of anesthesia administered. Management by a multidisciplinary team and attention to discharge planning are key aspects of care during and after orthopedic surgery.^[14–16]^

For critically ill patients, the American Association of Clinical Endocrinologists and the American Diabetes Association have recommended revising glucose targets precisely. In patients who are not critically ill, the recommendations are based on clinical experience and judgment. Hence, a reasonable general target for the hospitalized orthopedic patient undergoing surgery is 110 to 180 mg/dL. These goals should be flexible and individualized to the particular patient and the clinical circumstances, which sometimes can be difficult to conclude.^[15]^

The available data focus on increased risk for experience of complications or adverse events by patients with DM. However, there is a lack of literature containing analysis of continuous glycemia measurements before and after hospitalization. There are some reports that glycemic variability during postoperative period might also increase the risk of cardiovascular events or lead to more frequent periods of hypoglycemia, which might result in higher risk of losses of consciousness and falls, higher cardiovascular risk, disability, and mortality.^[16–23]^ However, few studies have reported on the effect of hypoglycemia during the perioperative period.^[24]^ Taking into consideration, that median length of hospital stay after TKA and THA is 3 days, the rehabilitation beginning on the day following the surgery may interfere with hypoglycemia or orthostatic hypotension. Patients at whom glycemia is not well controlled and despite are forced to perform rehabilitation program may develop debilitating consequences.

With the prevalence of this disease predicted to continue to increase and TKA, and THA being among the most common surgeries, it is important for orthopedic surgeons to consider glycemia in their plan of risk reduction for adverse postoperative events. If we consider the common risk factors of DM and OA, such as obesity, it seems that the number of patients having both diseases is threateningly big. According to the aforementioned literature, it is clear that the appropriate screening must be performed routinely. Depending on the results, administration of medications intra- and postoperatively has been shown to improve clinical outcomes. Individual patient characteristics and surgical case factors would be considered to choose the therapy. But to approach the patients with the individualized treatment modality, we must previously investigate the association of altered glucose status among them, thus based on the clinical studies. This factor remains the major rationale of the presenting study.

## 2. Materials and Methods

Patients for this prospective observational study will be recruited among those qualified for elective TKA or THA at the Department of Orthopaedics and Rehabilitation in academic Miedzyleski Specialist Hospital in Warsaw. 100 patients will be included. Every patient will be evaluated with the use of Finnish Diabetes Risk Score (FINDRISC), which allows for evaluation of risk of developing DM in 10 year time in patients who were not previously diagnosed^[25–27]^ and SCORE scale, which allows for evaluation of risk of death in 10 years due to cardiovascular events.^[28–31]^ To perform this evaluation data such as sex, age, body mass index (kg/m^2^), and waist circumference (cm) will be measured. Also, data such as physical activity, diet, used medications, smoking, and family history will be collected. Prior to the surgery every patient will undergo a blood withdrawal, which is routinely performed for every patient qualified for the orthopedic surgery. This blood sample analysis will include glycemia level, HbA1c, morphology, renal function parameters, lipidogram, level of C-reactive protein, and urine test. What is more, every patient will fulfill a questionnaire regarding functioning of the joint affected by OA. The scales used will be The Knee injury and Osteoarthritis Outcome Score scale (Knee injury and Osteoarthritis Outcome Score)^[32]^ or Harris Hip Score (HHS) scale^[33]^ according to the affected joint. Both scales contain questions about patients’ everyday life and limb function.

Inclusion criteria consist of willingness to participate in the study, being qualified for TKA or THA due to the primary OA and age >18 years.

Exclusion criteria consist of lack of acceptance to participate in the study, being qualified for nonsurgical treatment, other diseases but DM, compensated thyroid disorders and hypertension, poorly controlled DM and/or poorly controlled hypertension, patients with diabetes mellitus type 1, patients from the rest homes, pregnant women, patients who underwent lower limb surgeries recently, patients who in opinion of the research team might be poorly compliant, poor Polish language fluency.

Patients will have a glycemia measuring device (Dexcom G5, Inc., San Diego, CA) implemented twice. First time 14 days prior to the surgery, second time after the surgery and will keep wearing it for 14 days.

All patients will undergo TKA and THA procedures with standard perioperative antibiotics prophylaxis with use of 3 doses of 750 mg of cefuroxime–preoperatively, 6 and 12 hours postoperatively, and standard thrombosis prophylaxis of one dose of 40 mg low-molecular weight heparin. In every case posterior-stabilized total knee prosthesis (PERSONA, Zimmer Biomet, Warsaw, IN) or long-stem cementless Polarstem R3 total hip prosthesis (Smith & Nephew, Baar, Switzerland) will be used. Every participant will be rehabilitated immediately after the surgery with full weight bearing walking in the first 24 hours postoperatively, with the discharge from the hospital on the third day postoperatively.

During standard control visit, 14 days after the surgery skin sutures will be removed and the glycemia measuring device will be taken out. The measurements from the whole period of treatment will be collected from the device. Patients will be controlled during the planned visits 3, 6, 12, and 24 months after the surgery. In case of hyperglycemic event at any time of hospital stay or follow-up, patients will be referred to the internal medicine unit and remain controlled by the physicians according to the glycemic control guidelines.

During every visit glycemia level will be measured from standard blood samples, objective functional outcome will be assessed and radiological standard imaging of the operated limb will be performed. History of any cardiovascular event and/or surgical SSI will be noted.

The presenting study meets STrengthening the Reporting of OBservational studies in Epidemiology guidelines for ensuring high-quality presentation of the conducted prospective observational study.

## 3. Ethics, data management, and statistical analysis

All procedures used for this study have been approved by the Institutional Bioethics Committee of the Medical University of Warsaw (number of approval: KB/104/2020). The study was registered in the ClinicalTrials.gov database (NCT04484480). All patients will be obliged to give written consent for their participation in the study. Collected data will be stored at the Department in a database created particularly for this study and secured appropriately. In the database each participant will have a special code to minimize the risk of identification of a particular patient. Only patients who attend all visits, fill in all questionnaires and do not interrupt the course of the study in any other way will be included in the analysis. The consequence of the absence on a control visit will be exclusion from the study.

Power analysis was performed to assess how many patients should be included in the study. Determining effect size was hindered by lack of literature comparing mean values and standard deviations (SD) between groups with and without complications. The effect size can be deemed “large,” described numerically as 0.8 by Cohen. However, we decided to be more conservative and used an effect size of 0.6, located between “medium” and “large” in Cohen scale. Standard *P* value of .05, beta value of 0.8, and allocation ratio of 1/1 were used, resulting in a total needed sample size of 90 patients. Taking into consideration that 10% of patients might drop off the follow-up predicted number of participants is 100.

Basic analysis will include descriptive statistics of demographic characteristics to assess both groups homogeneity. Such analysis will be done several times during testing to detect any confounders that could impact the results. Continuous variables will be summarized with number of cases, means, standard deviations, medians, ranges, and first and third quartile (Q1 and Q3). All comparisons will be performed between continuous variables in paired groups, therefore either a 2-sided Student *t* test for paired groups or Wilcoxon Signed Rank test will be used. Distribution normality will be examined using the Shapiro–Wilk test. An overall alpha-level of 0.05 will be used as a cut-point for statistical significance and all statistical tests will be 2-sided. Significance level will be set at *P* value below .05. Outliers will be initially identified using interquartile range (IQR) as observations with values above Q3-1.5IQR or below Q1-1.5IQR and will be examined individually. All data will be presented with extreme and outlier observations or committed with appropriate annotation.

All statistical analyses will be conducted using SAS 9.4 for Windows (SAS Institute Inc., NC).

The study protocol was published before the start of the study on clinicaltrials.gov. The results of the study will be published in (inter-)national scientific journals and guidelines. The data that support the findings of this study will be available from the corresponding author - OA, upon reasonable request.

Data reported in the presenting study will be prospective observational data. The treatment will be reliable to the patients’ condition. And the procedure of implementing the glucose measuring device will be the same throughout the whole cohort. This has to be kept in mind for the interpretation of the results.

The inclusion and exclusion criteria presize how the authors deal with the risk of bias such as severity of illness or presence of comorbidities on intervention group assignment.

## 4. Outcomes measure

### 4.1. Primary outcomes

1. Changes of glycemia levels during 4-week perioperative period: mean glucose level during pre- and postoperative period, 24-hour glucose level amplitude, time of both higher and lower glucose level than average for the day in total, number of periods of both higher and lower than average glycemia levels for the day and its’ duration, number of days with higher mean glucose level than the mean for the corresponding pre- and postoperative period;
2. Any cardiovascular events during follow-up (heart stroke, brain stroke, coronary arteries disease, transient ischemic attack;
3. Any surgical site and/or implant complications during follow-up.

### 4.2. Secondary outcomes

1. Objective functional outcome in Knee Injury and Osteoarthritis Outcome Score (KOOS) or HHS scale and WOMAC;
2. Correlation of FINDRISC scale results with cardiovascular events and/or surgical site or implant complications.

### 4.3. Patient-reported outcomes

The KOOS was developed to evaluate short- and long-term symptoms and function in patients with knee injury. It is used for both OA assessment and several other types of knee injury.^[32,34]^ The questionnaire is currently culturally adapted in 39 languages, including a validated polish version.^[34–37]^

The HHS is a widely used and relatively simple multidimensional observational THA assessment.^[38]^ The questionnaire consists of questions about pain and activities of daily living, hip function, and range of motion. It has been used to differentiate the outcome of THA^[39]^ and for evaluating hip arthroscopy in younger patients.^[40]^ There is no polish language adaptation of HHS.

The WOMAC has been developed for knee and/or hip joints OA symptoms assessment. Reliability and validity in measuring changes in patients’ symptoms in affected joints has been extensively tested.^[41,42]^ There is no polish cultural adaptation of WOMAC, therefore WOMAC index will be calculated from KOOS.^[43]^

## 5. Risks

### 5.1. Cardiovascular risk

FINDRISC is widely used as a tool to recognize individuals at high risk of developing type 2 diabetes mellitus (T2DM) within 10 years. It has also been used to identify undiagnosed T2DM, abnormal glucose tolerance and metabolic syndrome.^[44,45]^

The SCORE is used to calculate the 10-year risk of cardiovascular mortality in individuals without preexisting atherosclerotic diseases. It has been designed to evaluate the impact of such risk factors as gender, age, smoking status, lipids, and systolic blood pressure, which significantly affect the overall risk and increase the likelihood of a future CVD event.^[46]^

## 6. Discussion

It is well established that DM in patients undergoing surgery is associated with increased risk for complications and increased length of hospital stay.^[45]^ In general, hyperglycemia is considered to cause abnormalities in the host’s physiologic response to a bacterial load not only in diagnosed with DM patients.^[46]^ Fasting blood glucose of > 140 mg/dL has been associated with a 3-fold increased risk for an infection independently on glucose metabolism.^[47]^

Periprosthetic joint infection remains a devastating complication after THA. Poor patient satisfaction and increased socioeconomic costs are associated with this along with increased mortality.^[48–50]^

Total joint arthroplasty is considered a relatively safe procedure globally for an overall condition, as reported rates of major cardiac complication, mostly attributable to myocardial infarction, are as low as 0.2% to 0.8%.^[51,52]^ However, this risk is prognosed to increase significantly with the predicted increase in number of arthroplasties in the future, therefore the adequate measures to minimize the influence on body metabolism, must be developed.^[53–55]^ Factors associated with cardiac complications are increased hospital mortality, increased length of stay and expenditure, increased number of noncardiac events.^[53,56–60]^

The literature lacks valuable information indisputably designing an evidence-based approach to patients suffering from T2DM in orthopedic surgery departments to decrease prevalence of potentially debilitating adverse events. Since there are currently available only empiric recommendations, there is a need for well-designed studies of preoperative T2DM management, particularly for orthopedic surgeries. This could minimize procedure delays, patients’ dissatisfaction and reduce morbidity and mortality. Hyperglycemia on the day of surgery is a significantly dangerous circumstance for both nondiabetic and T2DM patients, thus there is an urgency of developing the guidelines of proceeding on the patients presenting with this condition.^[61–63]^ The up-to-date database search utilized only a few papers investigating the role of preoperative glycemia on the postoperative outcomes.

A retrospective cohort study conducted by Robin et al aimed to investigate the impact of immediate preoperative capillary glucose levels on complications in a cohort of orthopedic patients admitted to undergo a TKA. This study presented no association between preoperative glycemia measured from capillaries and surgical complications or length of stay.^[64]^

Anderson et al observed that hyperglycemia provokes occurrence of 90-day deep SSI in orthopedic patients. Therefore, the glycemia on an admission may present as an important marker for SSI risk in orthopedics^[65]^ Arteaga et al found that the corrosion behavior on the implemented component of the knee joint prosthesis was not significantly different between diabetic and nondiabetic conditions of immersed disks or implant type. However, poor glycemic control facilitated aluminum release, which further elucidates implant failures. Those findings represent the hallmark of T2DM effects on SSI and implant colonization. Firstly, it is important to associate the presence of hyperglycemia with possibly increased risks and secondarily the design of specific surfaces should be considered. The induction of constructive regimen and healing responses in immunocompromised patients could enhance the outcomes in the risk group. Another finding included exhibition of 10% higher infection rates among T2DM patients than nondiabetic patients. This contributes to lack of efficiency in osseointegration and the issues on the molecular level at the implant–tissue adaptation.^[66]^

The heterogeneity of existing papers concerning the topic of T2DM complications in orthopedic surgery and a small abundance of research done underlines the priority to investigate the topic extensively. DM may paradoxically enhance hypoglycemic events, due to its difficult control in perioperative period. Roche et al demonstrate that the risk for revision TKA due to infection increases as glucose levels decline from the normal range. Preoperative fasting and antidiabetic medications may predispose patients to hypoglycemia.^[67]^ Because of this, several authors have advocated that scheduled surgeries should be performed during morning hours for diabetic patients.^[68–71]^ Prevention of both hyperglycemia and also hypoglycemia is important, as it has been associated with increased hospital costs, length of stay, greater amount of falls and higher mortality, poor outcomes, and more complications.^[67,70]^ The evaluation of their glycemic profile among the hospital stay is needed in order to establish a special protocol for treatment regimen in an effort to optimize the overall condition. And it remains an undiscovered topic. Since orthopedic surgery utilizes the general anesthesia, but afterwards patients are allowed to take a standing position rapidly, the proper control of blood glucose fluctuations are crucial in order to prevent falls, injuries, metabolic stress, and further complications of these processes. Therefore, the continuous glucose measure seems to be necessary to take a close look at that problem. In a retrospective review, Drews et al^[68]^ demonstrated that patients with low normal blood glucose, defined as 70 to 89 mg/mL, were at greater risk of developing perioperative hypoglycemia than hyperglycemic patients being treated with insulin; however, hyperglycemic insulin-treated patients were more likely to experience severe hypoglycemia. This evidences the priority of detailed control of glycemia throughout the patients in orthopedic units.

Previously undiagnosed diabetes or so-called hospital-induced stress hyperglycemia carries the same poor prognosis as known DM and should be treated.^[69]^ It is also important to establish a stable regimen of diet and pharmacologic therapy well in advance of anticipated discharge.^[72]^ Patients should be taught basic survival skills by the diabetes educator or nursing staff, particularly if they are new to glucose self-monitoring and insulin administration. Arrangements should be made for an office visit with the primary care physician within 2 weeks of discharge to review and adjust the treatment plan if necessary.

Adequate diabetes management during orthopedic surgery requires close attention to several factors: duration of diabetes, degree of glycemic control, presence and extent of complications, outpatient treatment regimen, and type of surgical procedure. In addition, unique patient situations demand individualization and flexibility of care. Poor acute phase perioperative glycemic control is associated with poor outcome. But the mechanism facilitating the effects are different among diabetic and nondiabetic patients, suggesting different glycemic management strategies for the 2 patient groups and underlying the need for detailed analysis of each patient.^[12]^ Therefore, the proper glycemic control reduces the occurrence of adverse outcomes during the perioperative period. Both hypoglycemia and hyperglycemia can prove to be detrimental and should be avoided by paying close attention to nutritional changes and to the antidiabetic treatment regimen, and by adhering to the general principles outlined above. A culture of optimal management fostered through awareness and education at all levels of surgical care in the hospital setting is vital to achieving and maintaining these objectives.

Results of the presenting paper will allow us to investigate the influence of orthopedic surgery on glycemia and possible modifications of hyperglycemia treatment in perioperative period in patients with T2DM or glucose intolerance. What is more, it will allow for investigation of changes of glycemia in patients with normal glycemia metabolism, potentially protecting them from hypoglycemia during hospital stay and increasing their awareness of potential development of DM in the future. Additionally, this study will correlate perioperative glycemic levels with risk of cardiovascular events in 1-year follow-up, and its influence on SSI and implant complications. Thanks to these findings surgeons will be able to lower the risk of such complications in the future, hence lowering mortality and increase the quality of life, especially in patients with DM and in those with high risk for developing this disorder. Another advantage of this study is the fact that it contains patients without recognized DM and without carbohydrate metabolism disorders and assesses their glycemia fluctuations. Such knowledge might help in development of an algorithm for perioperative care with stress on glucose level control as it is a period when patients are exposed to significant glycemia fluctuations. What is more, it must be emphasized that this study has a unique model, in which glycemia monitoring will involve both pre- and postoperative period for every included patient. Such a way of analyzing patients allows one to form a well visualizing control group, because every measurement after the surgery has its counterpart before the surgery. However this study did not exclude the disadvantages. Patients before the surgery and after hospital discharge will not be monitored all the time. Therefore, we must take into consideration the possible patients’ interference with glycemia levels in opposition to recommendations. That may lead to obtaining invalid results and risk of bias.

## Author contributions

**Conceptualization:** Artur Stolarczyk, Artur Mamcarz, Bartosz M. Maciąg

**Data curation:** Marcin Łapiński and Kuba Radzimowski

**Formal analysis:** Olga Adamska, Kuba Radzimowski and Maciej Świercz

**Investigation:** Artur Stolarczyk, Bartosz M. Maciąg and Piotr Stępiński

**Methodology:** Artur Stolarczyk, Olga Adamska, Bartosz M. Maciąg

**Project administration:** Artur Stolarczyk, Bartosz M. Maciąg

**Resources:** Olga Adamska, Marcin Łapiński

**Supervision:** Olga Adamska

**Validation:** Olga Adamska, Piotr Stępiński, Jakub Szymczak, Maciej Świercz and Krystian Żarnovsky

**Visualization:** Jakub Szymczak and Maciej Świercz

**Writing – original draft:** Artur Stolarczyk, Bartosz M. Maciąg and Piotr Stępiński

**Writing – review and editing:** Artur Stolarczyk, Olga Adamska, Bartosz M. Maciąg, Piotr Stępiński, and Krystian Żarnovsky.

**Conceptualization:** Artur Mamcarz, Bartosz M. Maciąg, Maciej Świercz, Marcin Łapiński, Olga Adamska.

**Data curation:** Artur Mamcarz, Bartosz M. Maciąg, Jakub Szymczak, Krystian Żarnovsky, Olga Adamska.

**Formal analysis:** Artur Mamcarz, Bartosz M. Maciąg, Jakub Szymczak, Krystian Żarnovsky, Olga Adamska, Piotr Stępiński.

**Funding acquisition:** Artur Stolarczyk.

**Investigation:** Bartosz M. Maciąg, Krystian Żarnovsky, Kuba Radzimowski, Maciej Świercz, Piotr Stępiński.

**Methodology:** Artur Mamcarz, Bartosz M. Maciąg, Jakub Szymczak, Krystian Żarnovsky, Kuba Radzimowski, Maciej Świercz, Olga Adamska.

**Project administration:** Artur Mamcarz, Artur Stolarczyk, Bartosz M. Maciąg, Jakub Szymczak, Maciej Świercz.

**Resources:** Bartosz M. Maciąg, Krystian Żarnovsky, Kuba Radzimowski, Marcin Łapiński.

**Software:** Artur Mamcarz, Artur Stolarczyk, Bartosz M. Maciąg, Jakub Szymczak, Krystian Żarnovsky, Kuba Radzimowski, Marcin Łapiński, Olga Adamska, Piotr Stępiński.

**Supervision:** Artur Mamcarz, Artur Stolarczyk, Bartosz M. Maciąg, Jakub Szymczak, Marcin Łapiński.

**Validation:** Artur Stolarczyk, Bartosz M. Maciąg, Olga Adamska, Piotr Stępiński.

**Visualization:** Artur Stolarczyk, Bartosz M. Maciąg, Krystian Żarnovsky, Kuba Radzimowski, Olga Adamska.

**Writing – original draft:** Bartosz M. Maciąg, Olga Adamska.

**Writing – review & editing:** Bartosz M. Maciąg, Olga Adamska.
